# Diarrhea and health inequity among Indigenous children in Brazil: results from the First National Survey of Indigenous People’s Health and Nutrition

**DOI:** 10.1186/s12889-015-1534-7

**Published:** 2015-02-27

**Authors:** Ana Lúcia Escobar, Carlos EA Coimbra, James R Welch, Bernardo L Horta, Ricardo Ventura Santos, Andrey M Cardoso

**Affiliations:** Departamento de Medicina, Universidade Federal de Rondônia, Rodovia BR-364 Km 9.5, Porto Velho, RO 76801-059 Brazil; Escola Nacional de Saúde Pública, Fundação Oswaldo Cruz, Rua Leopoldo Bulhões 1480, Rio de Janeiro, RJ 21041-210 Brazil; Programa de Pós-Graduação em Epidemiologia, Universidade Federal de Pelotas, Rua Marechal Deodoro 1160, Pelotas, RS 96020-220 Brazil; Departamento de Antropologia, Museu Nacional, Universidade Federal do Rio de Janeiro, Quinta da Boa Vista s/n, Rio de Janeiro, RJ 20940-040 Brazil

**Keywords:** Brazil, Indigenous peoples, Health surveys, Diarrhea, Social determinants, Child health

## Abstract

**Background:**

Globally, diarrhea is the second leading cause of death among children under five. In Brazil, mortality due to diarrhea underwent a significant reduction in recent decades principally due to expansion of the primary healthcare network, use of oral rehydration therapy, reduced child undernutrition, and improved access to safe drinking water. The First National Survey of Indigenous People’s Health and Nutrition in Brazil, conducted in 2008–2009, was the first survey based on a nationwide representative sample to study the prevalence of diarrhea and associated factors among Indigenous children in the country.

**Methods:**

The survey assessed the health and nutritional status of Indigenous children < 5 years of age based on a representative sample of major Brazilian geopolitical regions. A stratified probabilistic sampling was carried out for Indigenous villages. Within villages, children < 5 years of age in sampled households were included in the study. Interviews were based on a seven day recall period. Prevalence rates of acute diarrhea were calculated for independent variables and hierarchical multivariable analyses were conducted to assess associations.

**Results:**

Information on diarrhea was obtained for 5,828 children (95.1% of the total sample). The overall prevalence of diarrhea was 23.5%. Regional differences were observed, with the highest rate being in the North (38.1%). Higher risk of diarrhea was observed among younger children and those who had less maternal schooling, lower household socioeconomic status, undernutrition (weight-for-age deficit), presence of another child with diarrhea in the household, and occurrence of upper respiratory infection.

**Conclusions:**

According to results of the First National Survey of Indigenous People’s Health and Nutrition, almost a quarter of Indigenous children throughout the country had diarrhea during the previous week. This prevalence is substantially higher than that documented in 2006 for Brazilian children < 5 years generally (9.4%). Due to its exceedingly multicausal nature, the set of associated variables that remained associated with child diarrhea in the final multivariable model provide an excellent reflection of the diverse social and health inequities faced by Indigenous peoples in contemporary Brazil.

## Background

Among infectious and parasitic diseases, diarrhea contributes importantly to overall disease burden, especially in settings marked by socioeconomic inequality, poor sanitation, and inadequate public health services coverage. Globally, diarrheal disease is the second leading cause of death among children under five and every year approximately 800,000 deaths are related to diarrhea in this age group [1-3].

In Brazil, mortality due to diarrhea underwent a significant reduction during the last three decades principally due to expansion of the primary healthcare network, widespread use of oral rehydration therapy, reduced child undernutrition, and improved sanitation conditions largely associated with improved access to safe drinking water [4-7]. Among children under one year, the mortality rate fell about 90%, from 11.7 deaths per 1,000 live births in 1980 to 1.5 in 2005 [6].

Despite the progress achieved nationally with regard to child health generally, important differences persist between different population segments with respect to conditions affecting vulnerability to diarrhea [8]. For example, basic health and sanitation services coverage have improved for the general population, but Indigenous peoples in Brazil suffer from significant accumulated deficits in access to services such as clean water, sewage treatment, and primary healthcare as compared to the non-Indigenous population [9-11]. Studies carried out in different regions of Brazil have reported that infectious and parasitic diseases are responsible for 20 to 40% of hospital admissions among Indigenous children under five years of age and diarrhea may account for up to 90% of total reported admissions in this group depending on region and ethnicity [12-15].

Numerous studies globally have pointed to the persistence of large health disparities between Indigenous and non-Indigenous peoples even in countries, such as Australia, the United States, and Canada, which experienced significant social advances and health indicator improvements long before Brazil. Despite differences in scale, being Indigenous in these countries is also accompanied by poorer socioeconomic and health indicators [16-18]. In the case of Indigenous peoples in Brazil and other Latin American countries, infectious diseases, such as diarrhea, that are preventable by means of basic health measures predominate in peoples’ experiences of illness and deaths in diverse communities. Therefore, its confrontation by means of adequate political policy formulation and better healthcare services coverage and quality is imperative.

The aim of this paper is to analyze the prevalence of reported diarrhea and associated factors among Indigenous children under 5 years of age in Brazil, based on data from the First National Survey of the Health and Nutrition of Indigenous Peoples in Brazil (henceforth, “National Survey”), conducted in 2008–2009. This was the first study of its kind to include a nationwide representative sample of Indigenous peoples and aims at contributing to the understanding of the contemporary health profile of the nation’s Indigenous population in terms of diverse demographic, socioeconomic, and environmental factors.

## Methods

The National Survey assessed the health and nutritional status of Indigenous children < 5 years and women 14 to 49 years in Brazil. A representative sample of villages located in federally recognized Indigenous reserves was obtained by multi-stage probabilistic sampling stratified by geopolitical region, as previously reported [10]. The final sample included 123 villages distributed between the following regions: North (65 villages), Central-West (14), Northeast (23), and South/Southeast (21). Households in selected villages were investigated by either census or sample depending on estimated village population. In selected households, mothers, fathers, or other relatives of all children < 5 years of age were interviewed [10]. The respondent was asked whether the child had acute diarrhea during the seven days prior to the interview [19]. If the answer was affirmative, the respondent was also asked if the child received homemade or commercial oral rehydration therapy. The National Survey methodology also included a specific set of questions on feeding of children ≤ 24 months. Additional information about variables employed and construction of nutrition, morbidity, sanitation, and socioeconomic indicators are available elsewhere [10,20,21].

In initial data analysis, prevalence rates of diarrhea were calculated according to independent variables (region, demographic and socioeconomic variables, characteristics of the household environment, and maternal and child characteristics). Measures of associations were expressed as prevalence ratios (PR) and their corresponding 95% confidence intervals. Chi-square tests for heterogeneity and Student’s t-tests for linear trend were used to evaluate differences in proportions. Estimates were corrected for the complex sampling design of the study in order to obtain a stratified probabilistic sample whose primary selection unit was village and the weights of each participant were based on the relative size of each stratum and the proportion of women and children in the target population.

The multivariable analysis employed Poisson regression with robust adjustment of variance with presence of diarrhea during the previous week as the outcome variable. This analysis included only variables collected for all children < 5 years of age. Thus, variables related to feeding practices were not included because they were collected only for children < 24 months. Independent variables entered the analysis according to hierarchical level in the model [22,23]. All estimates were adjusted by region. The first and most distal level of the model included sociodemographic characteristics of the child (sex and age), a household goods index, presence of regular income, and maternal schooling. The second hierarchical level comprised variables related to household physical conditions (type of flooring, walls and roofing), household size and composition, and sanitation conditions (source of drinking water, location used to defecate, and presence of garbage collection in the village). The third level included maternal characteristics (age and parity). The fourth level encompassed child variables (place of delivery, birth weight, and nutritional status). Finally, the fifth and most proximal level included child’s hospitalization due to diarrhea during the preceding 12 months, presence of another child in the household with diarrhea in the prior week, and child’s acute lower respiratory infection in the preceding week.

Vaccination status against rotavirus was evaluated for inclusion in the model differently than other variables because the anti-rotavirus vaccine was only introduced in the Brazilian public health system in 2006 and, consequently, children 4 to 5 years of age in the sample had not had the opportunity to receive it during their first 6 months of age. The National Immunization Program of the Brazilian Ministry of Health stipulates that children receive the first dose at two months and the second at four months. Thus, diarrhea prevalence rates were compared for the subset of children 6 to 24 months according to vaccination status.

Multivariable analysis was conducted employing a backward selection procedure with p < 0.20 for initial selection at each level, controlling for region and variables retained in previous levels, and p < 0.05 for retention in the final model. This multivariable analysis procedure has been described in previous studies reporting on the National Survey [20,21]. All analyses were conducted using STATA 10.0 (College Station, Texas, USA).

This study was approved by the National Committee on Research Ethics (Comissão Nacional de Ética em Pesquisa – CONEP) and the National Indian Foundation (Fundação Nacional do Índio – FUNAI). Upon arrival at each community, the research team held meetings with leaders and other community members, during which the objectives and procedures of the study were clearly presented. A Free and Informed Collective Consent form was presented and signed by leaders, as well as other community representatives. Any village, household, or guardian could decline to participate at any moment.

## Results

Information on diarrhea was obtained for 5,828 children (95.1% of the total sample). The seven-day prevalence of reported diarrhea was 23.5%. Regionally, the highest unadjusted prevalence rate was recorded in the North (38.1%), followed by the Central-West (21.2%), Northeast (19.5%), and South/Southeast (17.9%) (Table 1). Only the North region presented a rate (PR 2.12, CI_95%_ 1.56–2.88) which was different from that in the reference region (South/Southeast). Prevalence rates of diarrhea were higher among children 6 to 23 (PR 2.07, CI_95%_ 1.67–2.55) and 24 to 35 months of age (PR 1.43, CI_95%_ 1.16–1.75). Diarrhea was more frequent among children whose mothers had no schooling (PR 1.37, CI_95%_ 1.04–1.80) and 1 to 4 years of schooling (PR 1.41, CI_95%_ 1.16–1.72), as well as those living in households in the lower strata of the household goods index: 1st tertile (PR 1.55, CI_95%_ 1.25–1.93) and 2nd tertile (PR 1.39, CI_95%_ 1.15–1.68).

**Table 1:** **Prevalence rates of reported diarrhea during the previous week among Indigenous children < 5 years of age, with prevalence ratios and confidence intervals, according to sociodemographic (first level) characteristics**

**Characteristic studied**	**N** ^**†**^	**Prevalence (%)**	**Crude PR**	**CI 95%**
**Region**				
South/Southeast	853	15.86	1.00	Reference
Northeast	1297	19.48	1.09	0.74–1.58
Central-West	1279	21.15	1.18	0.86–1.61
North	2399	38.06	2.12	1.56–2.88
**Sex**				
Female	2845	22.72	1.00	Reference
Male	2983	24.13	0.94	0.85–1.05
**Child’s age (months)**			p = 0.000*	
0 to 5	628	17.37	1.00	Reference
6 to 23	1804	35.90	2.07	1.67–2.55
24 to 35	1126	24.78	1.43	1.16–1.75
36 to 59	2270	14.97	0.86	0.68–1.09
**Maternal schooling (years)**			p = 0.023*	
0	1037	24.56	1.37	1.04–1.80
1 to 4	2555	25.22	1.41	1.16–1.72
5 to 9	1469	22.78	1.27	1.04–1.55
≥10	767	17.91	1.00	Reference
**Regular income**				
Yes	2518	22.97	1.00	Reference
No	3542	23.89	1.03	0.89–1.19
**Household goods index (tertile)**			p = 0.000*	
1st	2236	27.60	1.55	1.25–1.93
2nd	2051	24.67	1.39	1.15–1.68
3rd	1541	17.77	1.00	Reference

First National Survey of Indigenous People’s Health and Nutrition, Brazil, 2008–2009.

PR: Prevalence ratio.

CI: Confidence interval.

^†^Maximum N for each category, which may vary between variables due to missing data.

*Student’s t-test for linear trend.

Breastfeeding was practically universal in the sample, having been reported for 97.5% (CI_95%_ 96.5–98.2) of children < 24 months (results not presented in table). Among children 6 to 23 months, only 34.0% (CI_95%_ 29.6–38.7) were exclusively breastfed during the first six months of life. Among children < 6 months who were reported to have breastfed, 28.1% (CI_95%_ 23.1–33.6) were also fed by bottle. In all regions of the country, the unadjusted frequencies of children < 24 months who had breastfed were over 95%. Whereas in the North region 43.8% (CI_95%_ 37.1–50.9) of children between 6 and 23 months were exclusively breastfed until 6 months of age, this rate was consistently lower in the other regions [28.5% (CI_95%_ 21.6–36.5) in the Central-West, 31.6% (CI_95%_ 23.7–40.8) in the Northeast, and 32.5% (CI_95%_ 24.2–42.1) in the South/Southeast].

Among children 6 to 23 months, the subset of our sample for whom the anti-retrovirus vaccine was made available by the national health system, only 45.2% (CI_95%_ 38.2–52.5) had received both doses, with wide variation between regions, from 22.5% (CI_95%_ 15.0–32.4) in the North to 60.7% (CI_95%_ 50.0–70.5) in the Central-West (results not presented in table). Bivariate analysis of diarrhea according to vaccination status revealed no significant differences between groups. For this reason, this variable was not included in the multivariable model below.

Among children < 5 years with diarrhea, only 55.9% (CI_95%_ 50.9–60.8) were reported to have received oral rehydration therapy, varying from 46.3% (CI_95%_ 36.5–56.3) in the Northeast to 65.1% (CI_95%_ 52.1–76.1) in the Central-West region (results not presented in table).

Table 2 presents unadjusted diarrhea prevalence rates according to household and environmental characteristics. The crude risk of diarrhea was higher among children living in houses with wood (PR 1.51, CI_95%_ 1.21–1.89) and dirt (PR 1.70, CI_95%_ 1.31–2.20) flooring, walls made of wood (PR 1.62, CI_95%_ 1.27–2.06) and thatch or clay (PR 1.64, CI_95%_ 1.30–2.06), and roofing made of concrete or corrugated zinc/asbestos sheets (PR 1.42, CI_95%_ 1.15–1.74). It was also observed that diarrhea was less frequent among children in houses with canvas, plastic, or other roofing (PR 0.76, CI_95%_ 0.57–0.99). Children living in larger households, both those with 5 to 8 (PR 1.18, CI_95%_ 1.03–1.37) and ≥ 9 residents (PR 1.60, CI_95%_ 1.38–1.87), and with more resident children < 5 years of age, including 2 to 3 (PR 1.26, CI_95%_ 1.12–1.43), and ≥ 4 (PR 1.73, 1.37–2.17) children, also presented greater risk of diarrhea. Sanitation conditions such as absence of a faucet in the house, non-availability of filtered drinking water, and less adequate treatment of human and household waste were shown to increase occurrence of diarrhea (PR and CI_95%_ are shown in Table 2).

**Table 2:** **Prevalence rates of reported diarrhea during the previous week among Indigenous children < 5 years of age, with prevalence ratios and confidence intervals, according to household and environmental (second level) characteristics**

**Characteristic studied**	**N** ^**†**^	**Prevalence (%)**	**Crude PR**	**CI 95%**
**Flooring**				
Ceramic or cement	2230	17.87	1.00	Reference
Wood	1485	30.37	1.51	1.21–1.89
Dirt	2113	26.97	1.70	1.31–2.20
**Walls**				
Brick	2032	17.82	1.00	Reference
Wood	2113	28.83	1.62	1.27–2.06
Thatch or clay	1131	29.17	1.64	1.30–2.06
Canvas, plastic, or other	552	21.41	1.20	0.93–1.56
**Roofing**				
Clay tile	1689	17.70	1.00	Reference
Concrete or corrugated zinc/asbestos sheets	2231	23.43	1.42	1.15–1.74
Wood or thatch	1856	33.18	0.58	0.30–1.14
Canvas, plastic, or other	52	13.61	0.76	0.57–0.99
**Number of household residents**			p = 0.000*	
≤4	1373	19.11	1.00	Reference
5 to 8	2868	22.65	1.18	1.03–1.37
≥9	1587	30.67	1.60	1.38–1.87
**Number of household residents < 5 years**			p = 0.000*	
1	1982	19.74	1.00	Reference
2 to 3	3458	24.95	1.26	1.12–1.43
≥4	388	34.05	1.73	1.37–2.17
**Predominant source of water used for drinking**				
Municipal system	432	15.13	1.00	Reference
Spring or artesian well	3188	22.22	1.47	1.13–1.78
Shallow well	518	24.01	1.59	1.13–2.24
River, lake, or reservoir	812	40.28	2.66	2.18–3.25
Others	878	23.82	1.57	1.24–1.99
**Presence of faucet**				
Inside the house	889	18.23	1.00	Reference
Outside the house	2849	22.23	1.22	1.01–1.49
Other	2090	30.02	1.65	1.33–2.04
**Filtered drinking water**				
Yes	920	18.71	1.00	Reference
No	4908	24.63	1.32	1.05–1.65
**Chlorinated, boiled, filtered, or decanted drinking water**				
Yes	2761	24.14	1.00	Reference
No	3067	22.91	0.95	0.79–1.14
**Predominant destination of human waste**				
Sewage disposal system	202	11.45	1.00	Reference
Septic system	985	21.61	1.89	1.32–2.69
Rudimentary pit latrine	2516	23.28	2.03	1.53–2.71
River, lake, or ocean	29	17.65	1.54	0.53–4.46
**Defecation location**				
Indoor household facility	839	17.98	1.00	Reference
Outdoor household facility	2973	23.41	1.30	1.01–1.68
Outdoors in the open or other	2016	26.58	1.48	1.13–1.94
**Predominant destination of household trash**				
Collected by removal service	1015	20.41	1.00	Reference
Buried, discarded, or burned in the village	4751	24.10	1.18	0.91–1.54
Discarded in a river, lake, or ocean, or other	62	37.59	1.84	1.12–3.04

First National Survey of Indigenous People’s Health and Nutrition, Brazil, 2008–2009.

PR: Prevalence ratio.

CI: Confidence interval.

^†^Maximum N for each category, which may vary between variables due to missing data.

*Student’s t-test for linear trend.

As shown in Table 3, whereas mother parity was not associated with occurrence of diarrhea, children of mothers < 20 years of age presented higher crude risk of diarrhea (PR 1.50, CI_95%_ 1.15–1.95).

**Table 3:** **Prevalence rates of reported diarrhea during the previous week among Indigenous children < 5 years of age, with prevalence ratios and confidence intervals, according to maternal (third level) characteristics**

**Characteristic studied**	**N** ^**†**^	**Prevalence (%)**	**Crude PR**	**CI 95%**
**Maternal age (years)**			p = 0.000*	
<20	769	28.50	1.50	1.15–1.95
20 to 29	3005	23.43	1.25	0.98–1.58
30 to 39	1615	21.97	1.16	0.89–1.49
≥40	439	19.48	1.00	Reference
**Number of children ever had**			p = 0.786*	
0 to 1	886	23.71	1.00	Reference
2 to 3	1926	22.60	0.96	0.79–1.16
≥4	3016	23.92	1.00	0.84–1.20

First National Survey of Indigenous People’s Health and Nutrition, Brazil, 2008–2009.

PR: Prevalence ratio.

CI: Confidence interval.

^†^Maximum N for each category, which may vary between variables due to missing data.

*Student’s t-test for linear trend.

Children born in villages presented higher risk of diarrhea (PR 1.58, CI_95%_ 1.37–1.82) than those born at hospitals and other health facilities (Table 4). No association was observed for birthweight. Also more likely to present diarrhea were children with weight-for-age z-score < −1 (PR 1.56, CI_95%_ 1.35–1.81), weight-for-height z-score < −1 (PR 1.44, CI_95%_ 1.17–1.77), and height-for-age z-scores < 0 and ≥ −1 (PR 1.24, CI_95%_ 1.01–1.53) and < −1 (PR 1.46, CI_95%_ 1.20–1.78).

**Table 4:** **Prevalence rates of reported diarrhea during the previous week among Indigenous children < 5 years of age, with prevalence ratios and confidence intervals, according to child’s health and nutritional (forth level) characteristics**

**Characteristic studied**	**N** ^**†**^	**Prevalence (%)**	**Crude PR**	**CI 95%**
**Birth place**				
Hospital, healthcare unit, or other	3428	19.89	1.00	Reference
Village	2400	31.26	1.58	1.37–1.82
**Birthweight (grams)**				
≥2500	3538	22.26	1.00	Reference
<2500	290	21.73	0.96	0.75–1.22
**Weight-for-age (z-score)**			p = 0.000*	
≥0	1867	19.64	1.00	Reference
<0 and ≥ −1	2206	22.57	1.14	0.97–1.34
< −1	1575	30.19	1.56	1.35–1.81
**Height-for-age (z-score)**			p = 0.000*	
≥0	927	18.15	1.00	Reference
<0 and ≥ −1	1355	21.98	1.24	1.01–1.53
< −1	3546	25.87	1.46	1.20–1.78
**Weight-for-height (z-score)**			p = 0.003*	
≥0	3885	22.22	1.00	Reference
<0 and ≥ −1	1545	24.88	1.12	0.99–1.27
< −1	398	31.12	1.44	1.17–1.77

First National Survey of Indigenous People’s Health and Nutrition, Brazil, 2008–2009.

PR: Prevalence ratio.

CI: Confidence interval.

^†^Maximum N for each category, which may vary between variables due to missing data.

*Student’s t-test for linear trend.

Several variables related to child health status were shown to be associated with diarrhea, including hospitalization due to diarrhea (PR 1.21, CI_95%_ 1.00–1.46) or to any other cause (PR 1.20, CI_95%_ 1.05–1.37) in the previous year (Table 5). Also, children with anemia (PR 1.62, CI_95%_ 1.41–1.85), with lower respiratory tract infection in the previous week (PR 2.01, CI_95%_ 1.74–2.38), and living with another child with diarrhea (PR 2.03, CI_95%_ 1.74–2.38) were more likely to present diarrhea.

**Table 5:** **Prevalence rates of reported diarrhea during the previous week among Indigenous children < 5 years of age, with prevalence ratios and confidence intervals, according to proximal child health (fifth level) characteristics**

**Characteristic studied**	**N** ^**†**^	**Prevalence (%)**	**Crude PR**	**CI 95%**
**Hospitalization due to acute respiratory infection during prior year**				
No	4737	22.39	1.00	Reference
Yes	425	27.99	1.21	1.00–1.46
**Hospitalization during prior year**				
No	4737	22.39	1.00	Reference
Yes	1072	27.61	1.20	1.05–1.37
**Anemia**				
No	2310	18.51	1.00	Reference
Yes	2830	29.54	1.62	1.41–1.85
**Another child at home with diarrhea**				
No	4403	19.38	1.00	Reference
Yes	1425	39.74	2.03	1.74–2.38
**Lower respiratory tract infection during prior week**				
No	4548	19.64	1.00	Reference
Yes	1262	37.97	2.01	1.78–2.27

First National Survey of Indigenous People’s Health and Nutrition, Brazil, 2008–2009.

^†^Maximum N for each category, which may vary between variables due to missing data.

**Figure 1:**
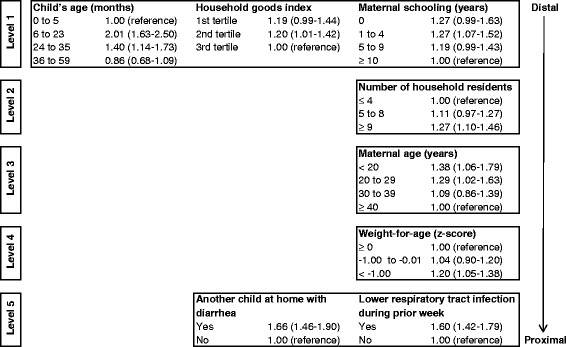
**Hierarchical model for diarrhea among children < 5 years of age, First National Survey of Indigenous People’s Health and Nutrition, Brazil, 2008–2009.** Values represent adjusted prevalence ratios with confidence intervals in parentheses. Geopolitical region was included as a control at all levels.

In the multivariable hierarchical analysis, variables from all levels remained associated with the occurrence of diarrhea in the final model: region, child’s age, household goods index, maternal schooling, number of household residents, maternal age, weight-for-age deficit, presence of another child with diarrhea in the household, and occurrence of upper respiratory infections (Figure 1).

## Discussion

In 2006, the prevalence of diarrhea among Brazilian children < 5 years, as reported by caregivers for a 15-day recall period, was 9.4% [24]. Contrasting this figure with the seven-day prevalence of the present study highlights the importance of diarrhea in the health profiles of Indigenous peoples in Brazil. According to results of the First National Survey of Indigenous People’s Health and Nutrition, almost a quarter of Indigenous children throughout the country had diarrhea during the previous week. This rate reached 38.1% in the North region. The degree of health inequity between Indigenous and non-Indigenous children is even more apparent when viewed diachronically. The prevalence of diarrhea among Indigenous children in 2008–2009 was still vastly higher than it was for the national population over a decade before (14.8% in 1996) [8]. In addition to this elevated frequency, analyses show that a wide range of demographic, socioeconomic, and heath variables were associated with the occurrence of diarrhea among Indigenous children.

The most vulnerable age group with respect to diarrhea was children between 6 and 23 months, who presented 101% greater chance of having had diarrhea during the previous week than infants < 6 months. This chance fell to 40% in the group of children between 24 and 35 months. The relatively lower chance of diarrhea among children < 6 months may be closely related to the protective effect of breastfeeding for infectious diseases in general and diarrhea in particular [25]. In culturally differentiated populations, such as Indigenous peoples, breastfeeding practices may differ from the national populations to which they pertain.

There are no epidemiological or systematic anthropological studies of breastfeeding practices among the Indigenous peoples of Brazil. However, among the diverse ethnographic accounts of these societies are indications that the practice is ubiquitous [26-28], which supports the National Survey results reported here. Nevertheless, although the practice appears to be nearly universal, several of its specificities as reported in the literature should be considered. Among these are observations that breastfeeding in many Indigenous societies in Brazil does not necessarily follow international health agency recommendations, including exclusive breastfeeding during the first six months of life, to reduce risk of disease and death due to diarrhea [25]. Many reports suggest that Indigenous children in Brazil may, during the first months of life, when they are also breastfeeding, receive solid foods, often premasticated, such as nuts, roasted corn, and pieces of meat, fruit, or bread. Among the Xavante, for example, Maybury-Lewis [29] noted that crying infants “…may be fed slops from the mouth of one of the women present. Grandmothers are particularly fond of feeding their tiny grandchildren in this way…” (p. 67). Similarly, Crocker [30] reported that among the Canela, “…depending on a mother’s attitude, she feeds her [2 to 4 month] baby mashes of foods, such as bananas, manioc, sweet potatoes, rice, and even brown sugar” (p. 158). Murphy and Murphy [31] observed similar baby feeding behavior among the Munduruku: “After the age of about four months, the child is fed gruels of tapioca and yam and somewhat later he is given meat, premasticated by the mother” (p. 194). The very act of breastfeeding also may be distinctive, since among many ethnic groups children may be breastfed by other lactating women in the family, such as their aunts and grandmothers [29,30].

Another practice that substantially interferes in child feeding is the use of bottles. The National Survey showed bottle feeding to be frequent and to occur early. Among Indigenous children < 6 months, 29.9% were bottle fed independently of whether or not they also breastfed at the same time. This finding is worrying since it occurs precisely during the most critical period of life – the first six months, when exclusive breastfeeding is indicated. The National Survey results do not permit identification of the liquids they receive by bottle, whether cow’s milk, baby formula, juice, or plain water. However, from the point of diarrhea transmission, administrating other foods during this critical phase of life not only compromises the child’s nutritional status and immune system development, but also favors the transmission of infectious agents by means of contaminated foods or liquids [32,33].

Diverse socioeconomic factors may be associated with risk of disease and death from diarrhea. In the case of the National Survey, maternal education and socioeconomic status remained statistically significant in the multivariable model, which finds ample support in several studies in Brazil and other countries showing these to be indicators of material resources availability and child care knowledge, especially with regard to diarrhea [34-37].

The results of the National Survey show a significant inverse association between the occurrence of diarrhea and maternal age, indicating a protective effect, particularly from the age of 40. Similar findings, widely reported in epidemiological studies in Brazil and elsewhere, are interpreted as reflecting the greater experience of older women in child care [38,39].

With respect to socioeconomic status, it is notable that diverse variables indicative of sanitation conditions and widely recognized as important in diarrhea control [36,40-43] were associated in the descriptive analyses with the occurrence of the disease among Indigenous children but were not retained in the final multivariable model. For example, children in households with a faucet, a toilet connected to a sewage system, drinking water that was filtered or derived from a public supply, or garbage collection service, were showed significantly less likely to have diarrhea. These variables were excluded from the model because they are highly correlated with the household goods socioeconomic index. Therefore, after controlling for socioeconomic condition, no association was observed for sanitation variables.

Undernutrition is among the most important health challenges for Indigenous children in Brazil, as amply demonstrated in several recent studies of specific communities and ethnic groups, as well as by the National Survey [20,44-49]. In the present study, results indicate that children with low weight-for-age showed over 20% higher risk of having diarrhea in the previous week. This finding is consistent with other research in which diarrhea figures among the infectious diseases that most affect growth and development of children < 5 years [50].

The interaction between diarrhea and undernutrition also severely compromises child immune functions, not only favoring the occurrence of new episodes of the illness, but also contributing to increased risk of other infectious diseases, such as pneumonia [50,51]. According to the National Survey, the risk of an Indigenous child having diarrhea in the previous week was 60% higher if he or she was also reported to have had pneumonia. There are several pathways that may explain the amplification of risks arising from the interaction between undernutrition, diarrhea, and acute respiratory disease. For example, a child may start with diarrhea and evolve towards acute malnutrition, finally contracting pneumonia, or, conversely, begin with a respiratory infection that worsens with an acute episode of diarrhea [51-55]. Whether occurring simultaneously (i.e., comorbidity) or as sequential infections, the interaction between diarrhea and pneumonia, aggravated by malnutrition, seriously endangers the lives of young children and contributes to the maintenance of high infant mortality rates in regions of the world marked by poverty, lack of access to basic public services, and food insecurity.

Another important aspect of diarrhea determination among Indigenous children was the association with number of household residents. Indigenous children living in households with ≥ 9 residents had 27% higher risk of contracting diarrhea than those in households with ≤ 4 inhabitants. At the same time, the risk of a child having diarrhea during the previous week increased 66% if another child in the house was also reported to have the disease during the same period. Although there is no consensus in the literature regarding the association between households with large numbers of inhabitants (overcrowding) and increased risk of diarrhea and pneumonia in children under five years, there is strong evidence pointing in this direction based on studies conducted in different context [56-58]. This potential association may derive from cosleeping practices among Indigenous peoples, which often differ from larger national populations.

Among the Bororo and Terena of Central Brazil, for example, practically all children < 2 years and about 80% of those under 10 cosleep with other family members [59,60]. According to Cohen [61], among the Kayapó-Xikrín, “…in an ideal arrangement, a toddler sleeps with her mother, while her brothers up to about 10 years sleep with their grandparents; older children sleep alone” (p. 69). Although it is not possible to generalize about the internal organization of Indigenous households nationally, child cosleeping is widespread. The anthropological literature on Indigenous peoples in Brazil is replete with references to houses lacking internal divisions and children sleeping together and with adults on the same bed or mat, as well as sharing hammocks [62,63]. Sleeping together in the same room or sharing the sleeping surface is an important cultural practice among numerous ethnic groups and is consistent with the associations observed in this study between number of residents and diarrhea risk. This point is based on the idea that pathogenic microorganisms are more easily transmitted to others in the same house, especially children, if living in close proximity to one another and under conditions of poor ventilation, dampness, and precarious sanitation installations.

In Brazil, diarrhea prevention and care for children are available through the public primary healthcare network and involve administration of anti-rotavirus vaccine and dispensation of oral rehydration therapy. Because the rotavirus vaccine was first made available through the Brazilian public health system in 2006, only children < 24 months in our sample were expected to have received the vaccine. In the age group 6 to 24 months, only 37.8% of Indigenous children nationally had received two doses of the vaccine. In the North, this proportion fell to 21.6%. The National Survey data show that at the time of fieldwork the proportion of Indigenous children who had received two doses of vaccine, as prescribed by the Health Ministry, was low and no protective effect was observed. Considering the importance of rotavirus in the determination of diarrhea among Indigenous children in Brazil [64-66] and positive evaluations of the vaccine reducing the incidence of diarrhea in the general Brazilian population [67,68], additional studies are necessary to evaluate its impact on diarrhea morbidity and mortality among Indigenous children.

The Brazilian Health Ministry adopted oral rehydration therapy for diarrhea management in 1982. Since then, its availability through the public health system has been a major factor responsible for the continued decline in infant mortality observed in recent decades [5,69]. The National Survey revealed that among Indigenous children reported as having had diarrhea in the previous week, 55.1% made use of oral rehydration therapy, whether homemade or industrialized. In the Northeast region, the frequency of use of oral rehydration therapy was lower (48.6%) than in the rest of the country, despite being the region in which Indigenous mothers had the highest number of years of schooling [10].

This study does not explain the low use of oral rehydration therapy in the management of diarrhea among Indigenous children. In addition to known deficiencies in healthcare services available to the Indigenous population in Brazil, including low coverage and quality [9,10,70], the potential influence of sociocultural factors in child diarrhea treatment also require attention. As described in several anthropological studies, the therapeutic program for any disease usually starts in the home and directly depends on interpretation of severity by family members, based on the patient’s exhibited signs and symptoms. The choice to initiate a child’s diarrhea treatment with homemade remedies or to seek rehydration salts through local health services depends on multiple factors, the analysis of which is beyond the scope of this study but certainly deserves to be investigated further [71-75].

This study has potential limitations that should be discussed. Analyses were based on data obtained through interviews with mothers, fathers, or other caregivers of Indigenous children under five years. Therefore, responses may have been influenced by local concepts and perceptions about diarrhea signs and symptoms, which vary among the country’s diverse ethnic groups [73,75,76]. Additionally, some non-parent relatives and other respondents may have lacked direct knowledge of information requested in interview questions. Although recall bias may have influenced responses, methodological studies have shown the quality of information on child diarrhea occurrence reported for a seven-day recall period to be adequate [19,77,78]. Also, the study design included only Indigenous peoples residing in federally recognized Indigenous reserves. This limitation deserves special note because many Indigenous peoples not contemplated, such as those living along roadsides, in makeshift camps, and in urban outskirts and slums, may live under even more precarious sanitation conditions. Finally, the cross-sectional design of this study does not allow conclusions about causality.

## Conclusion

The National Survey results reported here demonstrate that diarrhea weighs heavily in the epidemiology of Indigenous children in Brazil and highlight the magnitude of health inequity that persists in the country. The numerous variables found to be involved in the epidemiology of diarrhea reinforce the marginal position of this population segment within the national setting and reveal the broad linkages between social inequities deriving from a long history of political, social, and economic neglect, as well as impeded access to the marked infrastructural, health, socioeconomic, and educational improvements observed nationally in recent decades. These advances, coupled with expansion of the public healthcare network, have been widely recognized as responsible for the significant reductions in infectious and parasitic diseases, chronic malnutrition, and infant mortality observed among children nationally [6,7,34]. It should also be emphasized that improvements in basic sanitation and availability of safe drinking water in Indigenous villages, as well as the training of health workers are of fundamental importance in reducing the prevalence of diarrhea among Indigenous children in Brazil. Due to its exceedingly multicausal nature, the set of associated variables that remained associated with child diarrhea in the final multivariable model provide an excellent reflection of the diverse social and health inequities faced by Indigenous peoples in contemporary Brazil.

## Abbreviations

ABRASCO: Associação Brasileira de Saúde Coletiva
CONEP: Comissão Nacional de Ética em Pesquisa
CI: Confidence interval
PR: Prevalence ratio
FUNAI: Fundação Nacional do Índio
FUNASA: Fundação Nacional de Saúde
